# Before Helene’s Landfall: Analysis of Disaster Risk Perceptions and Preparedness Assessment in the Southeastern United States in 2023

**DOI:** 10.3390/ijerph22020155

**Published:** 2025-01-24

**Authors:** Young-Rock Hong, Haoran Chu, Zhigang Xie, Francis Dalisay

**Affiliations:** 1 Department of Family and Preventive Medicine, Emory University School of Medicine, Atlanta, GA 30322, USA; 2 Department of Health Services Research, Management and Policy, College of Public Health and Health Professions, University of Florida, Gainesville, FL 32610, USA; 3 Department of Public Relations, College of Journalism and Communications, University of Florida, Gainesville, FL 32611, USA; chu.h@ufl.edu (H.C.); f.dalisay@ufl.edu (F.D.); 4 Department of Public Health, University of North Florida, Jacksonville, FL 32224, USA; zhigang.xie@unf.edu

**Keywords:** extreme weather events, risk perception, disaster preparedness, Hurricane Helene

## Abstract

Hurricane Helene’s catastrophic impact on the Southeastern United States highlighted the critical importance of disaster preparedness. This study analyzes data from FEMA’s 2023 National Household Survey to examine pre-Helene disaster risk perception and preparedness levels among residents of six Southeastern states: Florida, Georgia, North Carolina, South Carolina, Tennessee, and Virginia. Our aim was to assess baseline preparedness and gain insights that could inform future disaster planning. The analysis revealed significant inter-state variations in risk perceptions, with Florida residents showing the highest awareness (84% believing a disaster was likely or very likely) and Virginia residents the lowest (63%). Perceived primary threats varied geographically, with hurricanes dominating concerns in coastal states (78% in Florida) and tornadoes in inland areas (68% in Georgia). Despite these differences, concerns about losing access to essential services during disasters were consistent across all states, with over 60% of residents extremely concerned about energy and food/shelter disruptions. While self-reported confidence in disaster preparedness was high across all states, there was a notable discrepancy between this confidence and residents’ estimated ability to manage without power or water. For instance, only 47% of Florida residents believed they could manage without power for more than one week despite their high-risk perception. Home or renters’ insurance coverage ranged from 65% in Florida to 77% in South Carolina. Hazard-specific insurance varied widely, with hurricane insurance coverage at 53% in Florida compared to about 12% in Tennessee. Our findings provide timely insights into the state of disaster preparedness in the wake of Helene, emphasizing more need for tailored, region-specific approaches to disaster preparedness and risk communication. The discrepancies between perceived and actual preparedness highlighted by this study can inform more effective strategies for enhancing community resilience in the face of increasing extreme weather events driven by climate change.

## 1. Introduction

Hurricane Helene made landfall in Florida’s Big Bend region as a Category 4 hurricane before causing catastrophic flooding across multiple Southeastern states in September 2024. Helene emerged as one of the most destructive storms in recent US history [1,2,3,4,5]. The hurricane’s unprecedented rainfall and flooding, particularly in western North Carolina, have been described as “biblical devastation” [3]. With at least 227 fatalities reported across six states in the Southeastern region—Florida, Georgia, North Carolina, South Carolina, Tennessee, and Virginia—and early estimates suggesting between USD 50 and 110 billion in total costs and economic loss, Helene’s impact has been both profound and far-reaching [1,4,5].

Climate scientists have established clear links between Hurricane Helene’s intensity and the effects of climate change [2,6,7]. A study by World Weather Attribution, a collaboration of academic researchers who study extreme weather events, found that climate change increased Helene’s rainfall by approximately 10% and made its winds about 13 mph (11%) stronger [6]. Helene formed over record-hot sea surface temperatures in the Gulf of Mexico, which have been attributed to human-caused climate change [2,6]. Research determined that human-caused climate change was responsible for almost half of the direct economic damages linked to Hurricane Helene [7]. These observations align with the broader scientific consensus on the increasing frequency and intensity of extreme weather events due to climate change. Recent studies indicate that warming ocean temperatures and increased atmospheric moisture content contribute to more intense hurricanes and heavier precipitation events [8]. Hurricane Helene’s devastating impacts, while reinforcing preparedness lessons learned from previous events like Hurricane Katrina, also revealed new challenges in the evolving landscape of climate-related disasters. Despite significant advances in disaster preparedness since previous major weather events, Helene’s unprecedented characteristics and impacts demonstrate the continuing need to understand and adapt our preparedness strategies [6,7,8].

To effectively manage the rising frequency of extreme weather events and enhance climate resilience, it is essential to improve public awareness and preparedness. These efforts can help mitigate impacts and ensure quick and appropriate responses. Beyond the conventional understanding that higher risk awareness and preparedness lead to reduced disaster risks and losses through emergency planning and evacuation, empirical studies underscore these benefits [9,10,11]. Research indicates that risk perceptions significantly influence preparedness behaviors [9], and that weather-related disaster insurance can improve recovery-related outcomes, including recovery time, economic stability, and satisfaction among those affected [11]. Studies have also found that a 1% increase in annual spending on risk preparedness and reduction can reduce damages by 0.21% [10]. Given this evidence supporting the importance of disaster preparedness, and in light of the impact of Hurricane Helene, there is a critical need to examine public perceptions of disaster risk and preparedness levels in the affected states.

Therefore, using data from the Federal Emergency Management Agency’s (FEMA) 2023 National Household Survey, this study explored how residents in Florida, Georgia, North Carolina, South Carolina, Virginia, and Tennessee perceived and prepared for disaster risks. While descriptive in nature, establishing this multi-state baseline of preparedness conditions is crucial for understanding regional variations in disaster readiness. By examining risk perceptions, preparedness actions, and potential barriers to preparedness among the residents of these states, this study seeks to provide valuable, timely insights for emergency management professionals, policymakers, and public health officials.

## 2. Methods

### 2.1. Data and Study Population

This study utilized data from the Federal Emergency Management Agency’s (FEMA) 2023 National Household Survey on Disaster Preparedness. This annual cross-sectional survey captures data on the disaster preparedness actions, attitudes, and motivations of the civilian, non-institutionalized US adult population (aged 18 years or older) with internet access, including residents of US territories [12]. The survey includes measures of preparedness actions, risk perceptions, disaster experience, and demographic characteristics, which have been used in previous studies to examine factors influencing household disaster preparedness. The survey was administered online in both English and Spanish between 1 February and 14 March 2023, with an oversample of American Indian, Alaska Native, Native Hawaiian, or Other Pacific Islander respondents to ensure an adequate representation of these populations [12]. For the present study, we focused on respondents from six Southeastern states affected by Hurricane Helene: Florida, Georgia, North Carolina, South Carolina, Tennessee, and Virginia (*n* = 1608). To ensure representativeness, survey weighting was applied using a statistical ranking procedure. Weights were calculated based on age, education, sex, race, disability status, homeownership, income, ethnicity, and geographic division, using population data from the US Census Bureau’s 2021 Five-Year American Community Survey and the 2020 Decennial Census of Island Areas [12]. This study did not require institutional review board approval because it used publicly available and de-identified data.

### 2.2. Measures

We employed a set of measures to assess disaster-related experiences, perceptions, preparedness levels, and communication expectations available in the FEMA National Household Survey. FEMA defined a disaster as an event that could threaten lives, disrupt public or emergency services like water and power, or damage property [12]. To assess disaster experience, participants were asked, “*Have you or your family ever experienced the impacts of a disaster?*” followed by inquiries about specific types of disasters encountered, including hurricanes, thunderstorms, tornadoes, and floods. Disaster risk perceptions were evaluated through two primary questions. Participants first rated the likelihood of a disaster impacting them, with response options ranging from “Very likely” to “Unlikely”. They then identified which types of disasters they perceived as having the greatest impact in their area, including hurricanes, power outages, floods, thunderstorms, and tornadoes. Preparedness was assessed through multiple measures. Participants rated their confidence in taking preparedness steps, answering the question, “*How confident are you that you can take steps to prepare for a disaster?*” with responses ranging from “Extremely confident” to “Not at all confident”. Resilience to utility disruptions was measured by asking how long respondents could stay at home without power and without running water, with options ranging from “Less than 1 day” to “More than 1 month”. We also used data on participants’ concerns about key community services during disasters, including worries about communications, energy (power/fuel), food/shelter, healthcare, community safety (crime), and transportation services. Lastly, we examined expected channels for receiving real-time disaster alerts and warnings from government sources, including federal, state, and local entities. Participants indicated their anticipated methods of receiving such communications, with options including email, app/social media, text/phone call, radio/TV, or no expected alerts from them.

### 2.3. Statistical Analysis

All analyses were conducted using weighted data to ensure the sample reflected a representative analysis of disaster preparedness for the US population. Descriptive statistics, including weighted percentages and 95% confidence intervals, were calculated for disaster-related experiences, perceptions, preparedness levels, and communication expectations. Wald Chi-square tests were performed to assess differences across states, with *p*-values reported to indicate statistical significance (at *p* < 0.05). All analyses were conducted using SAS 9.4 (SAS Institute, Cary, NC, USA) in September–October 2024.

## 3. Results

### 3.1. Sample Characteristics

The study sample comprised 1608 respondents from six Southeastern states: Florida (*n* = 978), Georgia (*n* = 164), North Carolina (*n* = 139), South Carolina (*n* = 105), Tennessee (*n* = 104), and Virginia (*n* = 118) (Table 1). Age distribution was relatively consistent across states, with the 18–29 age group representing the largest proportion in most states (ranging from 15.9% to 24.7%). Gender distribution was generally balanced, with females representing between 40.0% (South Carolina) and 52.1% (Georgia) of respondents. Respondents who self-identified as White comprised the majority in all states (59.6% in Georgia to 76.2% in Tennessee). The Black/African American representation ranged from 11.7% in Florida to 25.9% in Georgia. Hispanic/Latino ethnicity was most prevalent in Florida (29.2%) and least common in Tennessee (2.8%). Homeownership rates differed significantly across states (*p* = 0.0055), with the highest rates in Virginia (76.6%) and South Carolina (76.7%), and the lowest in Florida (63.7%). Home or renter’s insurance coverage also varied (*p* = 0.0131), ranging from 68.3% in Tennessee to 80.5% in Virginia. Regarding home types, single-unit homes without basements were the most common across all states, with the highest proportion in North Carolina (54.3%) and the lowest in Virginia (38.0%). Multi-unit apartment complexes or condos were most prevalent in Florida (26.8%) and least common in Tennessee (14.4%). Mobile or manufactured homes were most common in South Carolina (17.7%) and least common in Virginia (1.6%) (Table 1). Home or renter’s insurance coverage also varied significantly across states (*p* = 0.0131). Virginia and South Carolina reported the highest coverage rates at 80.5% and 76.9%, respectively, while Florida (69.0%) and Tennessee (68.3%) had the lowest rates.

### 3.2. Disaster Experience

**Figure 1:**
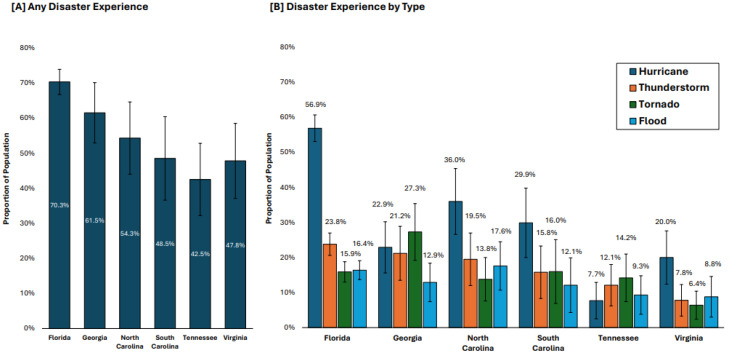
Prevalence of prior disaster experience (**A**) and disaster experience types (**B**) in 2023. Note: Error bars represent 95% confidence intervals.

Figure 1 shows variations in prior disaster experience across states (*p* < 0.001). Florida residents reported the highest rate of disaster experience at 70.3%, while Tennessee residents reported the lowest at 42.5%. By disaster type, hurricane experience was most common in Florida (56.9%) and least common in Tennessee (7.7%). Thunderstorm experience was also highest in Florida (23.8%) and lowest in Virginia (7.8%). Tornado experience peaked in Georgia (27.3%) and was lowest in Virginia (6.4%). Flood experience was relatively consistent across states, ranging from 8.8% in Virginia to 17.6% in North Carolina.

### 3.3. Disaster Risk Perceptions and Preparedness

**Figure 2:**
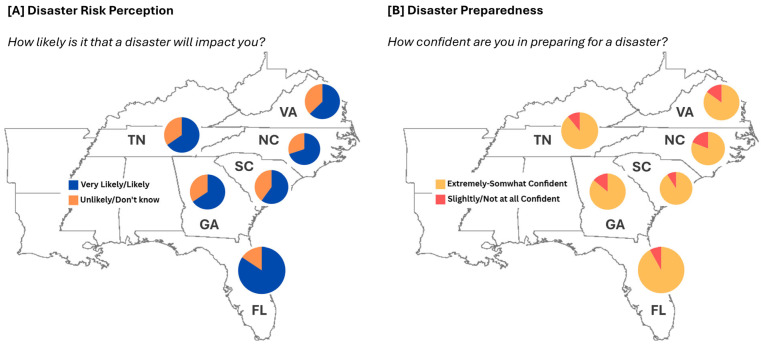
Disaster risk perceptions (**A**) and disaster preparedness confidence (**B**) across Southeastern states in 2023.

Figure 2 illustrates regional variations in disaster risk perception and preparedness confidence across six Southeastern states. Florida residents demonstrated the highest perceptions of disaster likelihood, with 84.4% believing a disaster was likely or very likely to impact them. In contrast, Virginia residents showed the lowest perception at 62.7%. The remaining states ranged from 63.9% in South Carolina to 70.0% in Tennessee. The types of disasters perceived as having the biggest impact differed significantly by state (*p* < 0.001; Table 2). Hurricanes were the top concern in coastal states, with 77.8% of Florida residents, 50.0% of North Carolina residents, and 56.4% of South Carolina residents identifying them as the biggest threat. Inland states showed different primary concerns; tornadoes were the main worry in Georgia (67.5%) and Tennessee (58.9%), while power outages were the principal concern in Virginia (50.9%). Confidence in disaster preparedness was generally high across all states, with no significant differences observed (*p* = 0.5093). Despite high confidence levels, the estimated ability to manage without essential services varied. The capacity to stay at home without power for more than 1 week ranged from 32.2% in Tennessee to 47.2% in Florida. Similarly, the ability to manage without running water for more than two weeks varied from 12.2% in Tennessee to 19.2% in South Carolina. Disaster-related concerns about key community services were consistently high across states. Concerns about energy (power/fuel) ranged from 54.9% in North Carolina to 64.3% in Virginia, while worries about communication services ranged from 42.3% in South Carolina to 57.7% in Florida (*p* = 0.0113).

### 3.4. Expected Disaster Alert and Warning Channels

**Figure 3:**
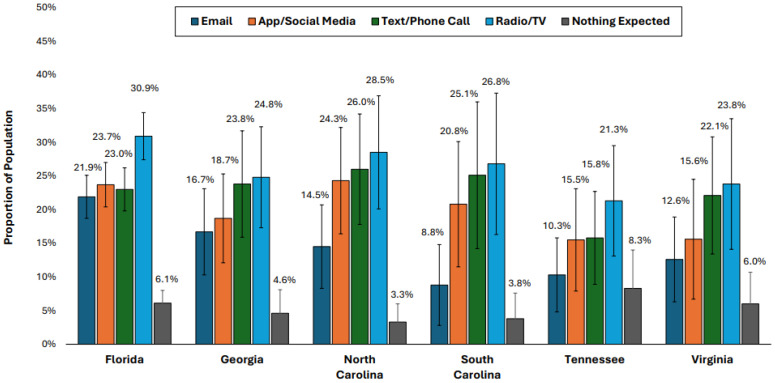
Expected ways of communication for real-time alerts and disaster warnings from federal, state, and local government sources in 2023. Note: Error bars represent 95% confidence intervals.

Figure 3 shows the expected ways of receiving real-time alerts and disaster warnings from government sources. The radio/TV was the most expected method, with the highest proportion in Florida (30.9%) and the lowest in Tennessee (21.3%). Text/phone calls were the second most common expectation in most states, ranging from 15.8% in Tennessee to 26.0% in North Carolina. App/social media notifications showed some variation, with the highest expectation in North Carolina (24.3%) and the lowest in Tennessee (15.5%). Interestingly, email alerts were expected mostly in Florida (21.9%) and the least in South Carolina (8.8%). Notably, a small proportion of respondents in each state did not expect to receive any alerts, ranging from 3.3% in North Carolina to 8.3% in Tennessee.

## 4. Discussion

Using data from FEMA’s 2023 National Household Survey, our findings showed regional variations in disaster risk perceptions, preparedness, and communication preferences across six Southeastern states. While overall confidence in preparedness for a disaster was high across the states, there were notable differences between perceived preparedness and estimated ability to manage without essential services (power, running water) during a disaster. Risk perceptions varied widely by state and geography, reflecting traditional hazard patterns: coastal states like Florida showed the highest perceptions of disaster risk, particularly for hurricanes (77.8%), while inland states like Tennessee and Georgia focused more on tornado risks (58.9% and 67.5%, respectively). However, these geographic variations revealed some paradoxical findings—for instance, while only 20% of Georgia respondents felt a disaster would impact them, 30% identified hurricanes as potentially having the biggest impact in their area. The observed differences in risk perception likely stem from regional climate patterns and past disaster experiences, which shape residents’ sense of vulnerability. However, as climate change intensifies, these traditional risk perceptions may leave populations unprepared for emerging threats, as seen in the unexpected impacts of Hurricane Helene in previously lower-risk areas like South Carolina, Tennessee, and Georgia.

Our findings also revealed concerning disparities in hazard-specific insurance coverage across states that became particularly consequential during Hurricane Helene’s unprecedented inland impacts [1,2,3,4,5]. While Florida shows the highest hurricane insurance coverage (53.2%), reflecting traditional coastal risk perceptions, inland states have notably lower rates (11.5% in Tennessee and 19.9% in Georgia). This insurance gap proved devastating during Helene, especially in Western North Carolina where only 1 in 200 homes had flood insurance despite this region ultimately suffering some of the storm’s worst impacts [13,14]. Although our analysis indicates flood insurance rates ranging from 25.2% in Tennessee to 35.2% in Virginia, these figures likely overestimate actual coverage in the most vulnerable inland areas [14]. The pattern of insurance coverage appears to follow traditional rather than emerging risk patterns—inland states have higher rates of tornado insurance (Georgia 32.3%, Tennessee 31.6%) compared to coastal states (Florida 18.8%, Virginia 14.5%), even as climate change increases their vulnerability to hurricane impacts. This misalignment between insurance coverage and evolving risk patterns is particularly problematic given recent research indicating that tropical cyclones are maintaining their intensity further inland due to climate change [2,15]. Helene’s severe inland impacts underscore the urgent need for expanded insurance coverage and risk communication strategies that better reflect these changing patterns of hurricane behavior and inland vulnerability. Additionally, the current risk-based premiums have made natural hazard insurance less affordable, which in turn reduces demand for this coverage [16]. This may largely explain the overall low uptake of natural hazard insurance across all states, even in areas with high disaster risk perceptions. This situation calls for policy changes, such as implementing a public–private partnership insurance system and introducing requirements to align with emerging risk patterns.

Prior disaster experience appears to influence risk perceptions and preparedness [17]. Florida, with the highest rate of prior disaster experience, also showed the highest risk perceptions. This underscores the potential role of education and awareness campaigns in areas with less disaster experience to help residents better understand and prepare for risks. Concrete steps for effective campaigns should include targeted education on specific regional risks and simulated disaster scenarios, alongside further formative research to explore the nuanced intentions, perceived limitations, and key determinants of residents’ preparedness. Concerns about disruptions to critical services were consistently high across all states, indicating a need for enhanced communication and planning around the continuity of essential services during disasters. For instance, during the days after Hurricane Helene, there were outages in cell phone service in North Carolina, which may have delayed the response times for residents needing assistance [18]. This highlights the need to plan around potential disruptions in communication services.

Moreover, although the expected channels for real-time disaster alerts and warnings varied by state, radio/TV was the most preferred. These results suggest that radio/TV could be used to disseminate real-time disaster alerts and warnings; however, there is also a need for a multi-channel approach to emergency communications tailored to each state’s preferences and demographics [19]. These findings also point to the potential value of leveraging interpersonal communication channels, especially in underserved and underrepresented communities, where residents often rely on close social networks, or bonding social capital, during crises [19,20]. Interpersonal channels could effectively complement gaps in access to official disaster information, ensuring that critical updates reach individuals who may have limited connectivity or access to governmental communication channels [20].

Despite our study showing relatively high disaster risk perceptions and confidence in preparing for disasters across these Southeastern states, actual preparedness regarding power and water supplies remained low. This underscores the need to enhance community preparedness, especially given the increasing scale of weather-related disasters in recent years [6,7,8], which have resulted in more severe damage and longer recovery times, including for water and power restoration. Current disaster preparedness guidelines from government agencies typically recommend one week of emergency supplies [21], but these may need to be updated to better align with emerging patterns in extreme weather events. Furthermore, efforts to improve disaster preparedness should leverage the experiences of communities with high disaster exposure to help inform and motivate preparedness in less-affected areas [22]. Planning for the continuity of critical services during disasters should be a high priority [23], given the consistent concerns across all states. Emergency alert systems should be designed with redundant communication channels, recognizing that modern communication infrastructure is highly vulnerable during disasters; power outages can disable cellular networks, internet services may become unavailable, and emergency response communications may overwhelm existing systems. While our study shows preferences for specific communication channels, the reliability of these channels during actual disasters cannot be guaranteed, emphasizing the importance of maintaining multiple ways to receive emergency information. There may be a need for increased promotion and accessibility of hazard-specific insurance coverage [11], particularly in high-risk coastal areas.

### Limitations

This study has several important limitations that should be considered when interpreting the results. First, as this study relies on secondary data from FEMA’s National Household Survey, we acknowledge the inherent constraints of using pre-existing survey data and conducting descriptive analyses. While FEMA’s survey provides standardized, nationally representative data collected through rigorous probability sampling methods, our analysis is limited to the questions and measures included in the original survey instrument. Our descriptive analytical approach, while appropriate for establishing baseline conditions and documenting preparedness patterns, does not allow us to make causal inferences about relationships between variables or test specific hypotheses about preparedness behaviors. The data were collected through an online web-based survey, which excluded adults without internet access. This sampling approach may have led to selection bias by underrepresenting populations with limited internet access, particularly in rural areas or among lower-income households. The survey was also only available in English and Spanish, potentially limiting participation from residents who primarily speak other languages. Second, the timing of data collection in 2023 represents a snapshot of preparedness and risk perceptions before Hurricane Helene. While this provides valuable baseline data, it does not capture potential changes in preparedness behaviors or risk perceptions that may have occurred in response to hurricane forecasts or other events between the survey period and Helene’s landfall. Third, the study relies on self-reported data, which may be subject to social desirability bias and recall bias. Respondents might have overestimated their preparedness levels or underreported their concerns to present themselves more favorably. Additionally, recall of previous disaster experiences may be influenced by the passage of time and the severity of impacts experienced. Lastly, while the survey included questions about expected communication channels for disaster alerts, it did not assess the reliability or effectiveness of these channels during actual disasters. This limits our ability to make recommendations about optimal communication strategies during emergency situations. Despite these limitations, our study provides a timely and comprehensive descriptive analysis of disaster preparedness in the Southeastern US, offering valuable insights for understanding pre-disaster community readiness. Despite these limitations, our findings provide crucial baseline data that can inform immediate efforts to enhance community resilience. This is particularly valuable given the increasing frequency and intensity of climate-related disasters in the Southeastern regions, as devastatingly demonstrated by Hurricane Helene’s impact.

## 5. Conclusions

As Hurricane Helene demonstrated, the Southeastern United States faces significant and evolving disaster risks in the era of climate change. Our findings show both strengths and vulnerabilities in the region’s disaster preparedness landscape. While many residents expressed confidence in their preparedness, there are concerning gaps between perceived and actual readiness, particularly for extended disruptions to essential services (communication, power). Geographic variations in risk perceptions and preparedness highlight the need for tailored, localized approaches to disaster risk communication and planning. Future research should examine how the gap between perceived and actual preparedness shifts over time through longitudinal studies, particularly investigating changes before and after major disasters like Hurricane Helene and Milton.

## Figures and Tables

**Table 1:** Characteristics of participants among six Southeastern states in 2023.

	Weighted % (95% CI) a						
Characteristics	Florida (*n* = 978)	Georgia (*n* = 164)	North Carolina (*n* = 139)	South Carolina (*n* = 105)	Tennessee (*n* = 104)	Virginia (*n* = 118)	*p*-Value
**Age Group**							0.8410
18–29	23.4 (19.9–26.9)	21.1 (13.7–28.6)	24.7 (15.0–34.4)	15.9 (6.8–25.1)	21.4 (13.2–29.7)	19.5 (10.2–28.8)	
30–39	14.8 (12.3–17.3)	18.4 (12.1–24.6)	14.5 (8.5–20.5)	10.9 (5.4–16.5)	13.0 (5.7–20.2)	16.3 (7.6–25.1)	
40–49	15.4 (12.6–18.2)	15.9 (8.5–23.3)	9.0 (3.3–14.6)	16.2 (6.1–26.3)	15.7 (7.5–23.9)	17.3 (9.4–25.1)	
50–59	14.2 (11.6–16.8)	15.9 (9.3–22.5)	14.6 (7.9–21.3)	17.9 (9.5–26.4)	20.9 (11.9–30.0)	15.2 (7.9–22.6)	
60–69	13.4 (11.0–15.8)	16.0 (9.9–22.2)	18.8 (10.9–26.7)	14.5 (6.9–22.1)	12.5 (5.6–19.4)	13.7 (7.3–20.1)	
70+	18.8 (15.9–21.7)	12.6 (7.4–17.9)	18.4 (10.4–26.3)	24.5 (14.0–35.0)	16.5 (9.4–23.7)	18.0 (10.0–25.9)	
**Sex**							0.6020
Female	51.1 (47.3–54.9)	52.1(43.3–60.9)	50.4 (40.3–60.6)	40.0 (28.9–51.2)	50.7 (40.2–61.3)	41.2 (30.4–52.1)	
Male	48.4 (44.6–52.2)	46.9(38.1–55.7)	49.2 (39–59.4)	56.3 (44.8–67.9)	47.4 (36.9–58.0)	58.2 (47.3–69.0)	
Other/non-binary	0.5 (0.1–0.9)	0.9 (0.0–2.8)	0.4 (0.0–1.1)	3.6 (0.0–9.2)	1.9 (0.0–4.0)	0.6 (0.0–1.7)	
**Race**							0.0096
Black/African American	11.7 (9.3–14.2)	25.9 (17.8–33.9)	15.8 (9.4–22.3)	15.7 (7.7–23.8)	17.4 (10.1–24.6)	12.5 (6.4–18.6)	
Other race b	13.1 (10.2–16.0)	14.5 (7.6–21.3)	16.7 (7.2–26.1)	8.9 (2.1–15.7)	6.4 (0.5–12.2)	22.8 (12.7–32.9)	
White	75.2 (71.7–78.6)	59.6 (50.7–68.6)	67.5 (57.4–77.5)	75.4 (65.5–85.2)	76.2 (67.5–85.0)	64.7 (54.1–75.3)	
**Ethnicity**							
Hispanic/Latino	29.2 (25.9–32.5)	10.5 (4.3–16.7)	3.5 (0.4–6.6)	5.1 (0.0–11.0)	2.8 (0.2–5.3)	10.7 (3.8–17.6)	
Non-Hispanic/Latino	70.8 (67.5–74.1)	89.5 (83.3–95.7)	96.5 (93.4–99.6)	94.9 (89.0–100)	97.2 (94.7–99.8)	89.3 (82.4–96.2)	
**Education**							0.0826
High school or less	38.9 (35.0–42.8)	34.3 (25.4–43.1)	37.2 (26.5–47.8)	34.2 (22.2–46.1)	44.8 (34.2–55.5)	23.8 (13.4–34.1)	
Some college, no degree	19.9 (17.1–22.7)	21.7 (14.7–28.7)	21.6 (14.4–28.8)	19.1 (10.4–27.8)	27.1 (17.7–36.5)	18.1 (10.9–25.3)	
College degree	29.8 (26.6–33.0)	27.3 (19.9–34.8)	31.2 (22.3–40.0)	33.3 (23.1–43.5)	18.2 (11.3–25.2)	43.9 (33.3–54.4)	
Post graduate work/degree or professional degree	11.4 (9.1–13.7)	16.7 (10.2–23.3)	10.1 (4.3–15.9)	13.4 (4.4–22.5)	9.8 (3.4–16.3)	14.2 (6.3–22.1)	
**Having Disability**	14.1 (11.9–16.3)	13.2 (8.7–17.6)	15.3 (9.6–20.9)	21.8 (13–30.5)	12.4 (6.3–18.5)	14.4 (8.4–20.5)	0.6113
**No. Adults in Household**							0.1797
1	17.9 (15.1–20.7)	16.9 (10.5–23.3)	22.9 (14.7–31.0)	16.2 (9.4–23.1)	13.2 (6.8–19.5)	23.7 (15.8–31.6)	
2	48.7 (44.9–52.5)	47.6 (38.7–56.4)	49.6 (39.4–59.7)	62.8 (51.8–73.9)	58.3 (48.1–68.6)	49.6 (38.8–60.4)	
3	16.9 (14.1–19.6)	24.6 (16.2–33)	13.6 (7.2–20.0)	9.6 (3.6–15.6)	16.2 (8.3–24.1)	16.4 (8.0–24.7)	
4+	16.5 (13.6–19.5)	11 (5.7–16.2)	14.0 (5.1–22.8)	11.4 (2.7–20.0)	12.3 (5.9–18.6)	10.3 (3.8–16.8)	
**No. Children in Household**							0.7329
0	65.5 (61.9–69.1)	61.3 (52.3–70.3)	65.2 (54.8–75.6)	65.8 (54.3–77.4)	70.3 (61.1–79.4)	61.6 (50.6–72.5)	
1	16.6 (13.8–19.4)	18.0 (10–25.9)	17.3 (7.3–27.3)	12.3 (6.1–18.4)	9.6 (4.2–15.0)	12.7 (5.2–20.2)	
2	11.6 (9.1–14.1)	12.1 (6.1–18.2)	11.3 (5.4–17.1)	13.3 (3.9–22.7)	9.1 (3.5–14.7)	15.3 (7.4–23.2)	
3+	6.2 (4.4–8.0)	8.6 (3.8–13.4)	6.3 (2.0–10.5)	8.6 (0.9–16.3)	11.1 (4.8–17.4)	10.5 (2.0–18.9)	
**Living with Someone Requiring Assistance**	16.4 (13.6–19.2)	18.1 (11.7–24.5)	13.2 (7.0–19.4)	15.8 (7.8–23.9)	16.7 (9–24.4)	9.8 (4.7–14.9)	0.5987
**Household Income (USD)**							
Less than USD 25,000	17.9 (15.1–20.6)	12.9 (8.3–17.6)	16.3 (9.0–23.7)	17.3 (9.7–24.9)	23.4 (14.3–32.5)	14.9 (7.8–22.0)	0.0036
USD 25,000–49,999	23.6 (20.7–26.6)	11.9 (6.7–17.1)	18.3 (11.8–24.9)	23.1 (12.8–33.5)	22.1 (14.2–30.0)	12.6 (6.9–18.2)	
USD 50,000–99,999	29.1 (25.9–32.3)	28.9 (21.3–36.5)	33.7 (24.5–42.8)	19.9 (10.9–28.8)	32.1 (22.7–41.6)	29.6 (20.8–38.5)	
USD 100K+	29.5 (25.5–33.4)	46.2 (37.2–55.3)	31.7 (21.1–42.3)	39.7 (27.8–51.6)	22.4 (12.3–32.4)	42.9 (31.7–54.1)	
**Homeownership**							0.0055
Own	63.7 (60.1–67.3)	73.9 (66.1–81.8)	61.2 (51.1–71.4)	76.7 (67.1–86.3)	72.4 (63.2–81.6)	76.6 (68.1–85.1)	
Rent	36.3 (32.7–39.9)	26.1 (18.2–33.9)	38.8 (28.6–48.9)	23.3 (13.7–32.9)	27.6 (18.4–36.8)	23.4 (14.9–31.9)	
**Home Type**							<0.0001
Mobile home or manufactured home c	9.4 (6.8–11.9)	4.7 (1.8–7.7)	9.8 (4.8–14.7)	17.7(7.3–28.2)	9.7 (2.8–16.6)	1.6 (0.0–3.5)	
Multi-unit apartment complex or condo	26.8 (23.6–30.1)	18.8 (12.7–24.9)	17.4 (10.0–24.7)	18.3 (9.7–27.0)	14.4 (7.1–21.7)	22.0 (13.2–30.8)	
Single-unit home with a basement	7.3 (5.4–9.1)	30.4 (22.0–38.7)	13.5 (7.3–19.6)	12.1 (3.5–20.6)	25.5 (16.5–34.5)	36.3 (26.0–46.6)	
Single-unit home without a basement	52.7 (48.9–56.5)	41.4 (32.5–50.2)	54.3 (44.1–64.5)	47.6 (35.9–59.3)	47.6 (37.0–58.1)	38.0 (27.4–48.7)	
Other type	3.8 (2.4–5.2)	4.8 (0.8–8.7)	5.1 (0.0–13.1)	4.3 (0.1–8.4)	2.8 (0.0–6.6)	2.1 (0.0–4.7)	
**Having Home or Renter Insurance**	69.0 (65.3–72.6)	77.4 (69.8–84.9)	71.5 (61.4–81.5)	76.9 (67.0–86.8)	68.3 (58.3–78.4)	80.5 (71.5–89.5)	0.0131
**Separate Hazard-Specific Insurance**							
Fire	21.1 (18.1–24.2)	30.3 (22.1–38.6)	25.4 (16.6–34.3)	29.8 (18.3–41.2)	23.5 (14.5–32.6)	22.0 (14.2–29.7)	0.3103
Hurricane	53.2 (49.4–57.0)	19.9 (13.1–26.7)	28.9 (19.0–38.9)	38.5 (26.8–50.2)	11.5 (5.3–17.7)	23.1 (14.1–32.1)	<0.0001
Tornado	18.8 (15.8–21.7)	32.3 (24.0–40.7)	21.7 (12.5–30.9)	27.8 (16.2–39.4)	31.6 (21.9–41.3)	14.5 (7.5–21.5)	0.0035
Flood	34.8 (31.3–38.3)	33.5 (25.3–41.7)	28.1 (18.3–37.8)	28.2 (17.2–39.2)	25.2 (16.3–34.1)	35.2 (24.4–45.9)	0.3329
**Monthly Spending on Rent/Mortgage**							0.0094
USD 0	21.8 (18.5–25.1)	24.2 (16.0–32.3)	18.9 (10.7–27.2)	28.2 (17.2–39.2)	31.2 (21.5–41.0)	28.4 (18.6–38.3)	
USD 1–750	13.3 (10.9–15.7)	15.4 (9.6–21.2)	21.2 (14.1–28.3)	23.0 (13.5–32.5)	24.9 (15.7–34.0)	10.9 (5.9–16.0)	
USD 751–1000	11.4 (9.2–13.5)	8.6 (4.2–13.1)	11.6 (5.8–17.3)	9.4 (4.1–14.7)	12.3 (5.8–18.9)	17.1 (8.2–25.9)	
USD 1001–1500	16.9 (14.1–19.6)	20.8 (13.8–27.8)	16.0 (9.0–23.0)	11.4 (4.1–18.6)	16.6 (8.6–24.6)	11.0 (5.1–17.0)	
USD 1501–2000	15.2 (12.4–17.9)	10.8 (5.7–16.0)	13.5 (7.0–20.0)	7.2 (1.6–12.8)	4.7 (0.1–9.4)	14.1 (6.6–21.7)	
More than USD 2000	17.2 (14.2–20.1)	14.5 (7.8–21.2)	14.7 (5.1–24.3)	12.9 (3.8–21.9)	9.3 (3.0–15.6)	14.7 (6.7–22.7)	
Do not know	4.3 (2.6–6.1)	5.6 (1.5–9.8)	4.2 (0.2–8.1)	7.9 (0.8–15.0)	0.9 (0.0–2.6)	3.6 (0.0–8.1)	

a. Applied survey weighting to ensure the sample reflects a representative analysis of disaster preparedness for the US population. b. Includes American Indian, Alaska Native, Native Hawaiian/Pacific Islanders, and multiracial. c. Factory-built housing units constructed to federal Manufactured Home Construction and Safety Standards.

**Table 2:** Disaster risk perception, preparedness, and disaster-related worries about community services in 2023.

	Weighted % (95% CI) a						
	Florida (*n* = 978)	Georgia (*n* = 164)	North Carolina (*n* = 139)	South Carolina (*n* = 105)	Tennessee (*n* = 104)	Virginia (*n* = 118)	*p*-Value
**Disaster Risk Perceptions**							
***How likely is it that a disaster will impact you?***							<0.0001
Very likely	35.2 (31.6–38.8)	20.4 (13.8–27.0)	29.1 (19.0–39.2)	26.2 (16.5–35.9)	26.6 (17.5–35.7)	16.9 (9.7–24.2)	
Likely	49.2 (45.4–53.0)	45.3 (36.5–54.1)	40.9 (31.3–50.5)	33.7 (22.9–44.5)	38.8 (28.6–49.0)	45.8 (34.9–56.7)	
Unlikely	10.6 (7.9–13.4)	20.7 (13.7–27.7)	18.9 (11.7–26.0)	27.8 (16.1–39.5)	21.1 (11.8–30.3)	26.2 (17.0–35.4)	
Do not know	4.9 (3.5–6.3)	13.6 (6.4–20.8)	11.1 (3.6–18.6)	12.3 (4.4–20.2)	13.6 (6.5–20.7)	11.1 (4.8–17.4)	
***Types of disaster that would have the biggest impact on where you live***							
Hurricane	77.8 (74.5–81.1)	30.0 (22.0–38.0)	50.0 (39.8–60.1)	56.4 (44.3–68.5)	9.2 (3.7–14.6)	42.6 (32.2–53.1)	<0.0001
Power Outage	36.4 (32.7–40.0)	41.7 (32.9–50.6)	43.8 (34.1–53.6)	35.3 (24.6–46.1)	49.3 (38.7–59.8)	50.9 (40.2–61.7)	0.0418
Flood	47.1 (43.3–50.8)	30.0 (21.9–38.0)	47.8 (37.6–58.0)	31.7 (21.6–41.9)	32.0 (22.2–41.8)	31.8 (22.4–41.3)	<0.0001
Thunderstorm	45.7 (41.9–49.5)	54.7 (45.9–63.5)	46.6 (36.6–56.5)	43.3 (31.8–54.8)	52.3 (41.8–62.9)	33.4 (23.2–43.7)	0.0568
Tornado	43.0 (39.3–46.8)	67.5 (59.1–75.8)	57.5 (47.4–67.6)	57.7 (45.9–69.5)	58.9 (48.5–69.4)	39.6 (29.2–50.0)	<0.0001
**Disaster Preparedness**							
***How confident are you in preparing for a disaster?***							0.5093
Extremely confident	36.1 (32.5–39.8)	26.4 (18.0–34.8)	28.2 (19.4–36.9)	41.1 (29.3–53.0)	25.0 (15.9–34.1)	26.9 (16.4–37.4)	
Moderately confident	35.2 (31.7–38.7)	38.1 (29.5–46.7)	26.7 (18.4–35.0)	32.0 (20.7–43.3)	34.9 (24.7–45.0)	33.4 (24.0–42.9)	
Somewhat confident	20.6 (17.4–23.7)	21.5 (14.8–28.1)	26.1 (17.9–34.2)	18.3 (10.1–26.4)	29.1 (19.4–38.8)	25.1 (16.3–33.9)	
Slightly confident	5.0 (3.1–6.9)	6.8 (3.0–10.7)	7.6 (3.1–12.1)	3.7 (0.3–7.1)	5.0 (1.3–8.7)	10.4 (2.7–18.1)	
Not at all confident	1.6 (0.4–2.9)	3.0 (0.1–6.0)	3.7 (0.0–9.4)	2.2 (0.0–5.2)	3.5 (0.0–7.7)	2.6 (0.0–6.1)	
Do not know	1.4 (0.6–2.3)	4.2 (0.5–7.9)	7.8 (0.0–16.8)	2.6 (0.0–5.7)	2.5 (0.0–5.7)	1.6 (0.0–3.5)	
***How long could stay at home without power?***							0.0134
Do not know	7.1 (5.2–9.0)	9.6 (4.8–14.3)	17.5 (6.9–28.0)	8.3 (2.7–13.8)	13.0 (5.3–20.8)	5.7 (1.3–10.1)	
Less than 1 day	6.1 (4.2–8.0)	12.7 (5.7–19.7)	6.9 (2.2–11.6)	8.0 (1.4–14.6)	7.1 (1.6–12.6)	2.2 (0.0–4.8)	
1 to 3 days	15.5 (12.7–18.3)	9.7 (4.9–14.6)	17.7 (10.5–25)	16.2 (7.2–25.3)	25.5 (15.9–35)	20.3 (12.4–28.3)	
3 days to 1 week	24.1 (20.8–27.4)	25.4 (17.8–33.1)	23.6 (15.9–31.3)	12.4 (6.1–18.7)	22.3 (14.1–30.5)	26.4 (17.2–35.5)	
More than 1 week	17.2 (14.4–19.9)	16.9 (9.8–23.9)	14.4 (7.7–21.2)	25.7 (15.1–36.4)	14.2 (6.7–21.6)	18.2 (8.7–27.6)	
More than 2 weeks	13.0 (10.5–15.5)	14.4 (8.5–20.3)	8.3 (3.7–12.8)	11.6 (5.0–18.2)	6.3 (1.8–10.7)	9.4 (3.4–15.4)	
More than 1 month	17.0 (14.0–19.9)	11.3 (6.4–16.2)	11.6 (5.8–17.5)	17.8 (7.6–28.1)	11.7 (5.1–18.2)	17.8 (9.0–26.6)	
***How long could stay at home without running water?***							0.4247
Do not know	6.7 (4.9–8.6)	6.8 (2.5–11.2)	12.0 (4.0–19.9)	12.3 (4.7–19.9)	9.9 (2.8–16.9)	7.6 (0.9–14.3)	
Less than 1 day	12.3 (9.9–14.8)	16.8 (9.6–24)	13.4 (6.8–20.1)	16.4 (8.0–24.8)	17.5 (9.5–25.6)	11.5 (4.8–18.2)	
1 to 3 days	23.4 (20.1–26.6)	21.7 (14.3–29.1)	26.5 (18.1–34.9)	25.2 (15.0–35.3)	31.9 (22.0–41.8)	26.0 (16.7–35.3)	
3 days to 1 week	26.2 (22.8–29.6)	25.5 (17.6–33.5)	26.4 (16.9–35.9)	14.6 (7.6–21.6)	18.5 (10.7–26.3)	28.8 (19.0–38.7)	
More than 1 week	12.4 (10.0–14.9)	12.3 (6.8–17.8)	5.5 (1.9–9.0)	12.4 (5.1–19.6)	10.1 (3.8–16.4)	9.9 (3.9–15.9)	
More than 2 weeks	9.3 (7.1–11.4)	8.2 (3.8–12.6)	8.6 (3.2–14.1)	6.9 (2.0–11.8)	7.0 (2.2–11.7)	6.5 (1.8–11.1)	
More than 1 month	9.7 (7.5–12.0)	8.6 (4.5–12.7)	7.6 (2.7–12.5)	12.3 (1.6–22.9)	5.2 (0.6–9.7)	9.6 (3.1–16.2)	
**Disaster-Related Worries About Key Community Services**							
Communication service	57.7 (53.9–61.4)	56.5 (47.7–65.3)	44.6 (34.3–55.0)	42.3 (30.6–53.9)	51.2 (40.7–61.8)	53.9 (43.3–64.5)	0.0113
Energy (power/fuel)	64.2 (60.5–67.9)	60.4 (51.7–69.2)	54.9 (44.8–65.1)	58.0 (46.2–69.8)	60.3 (50.0–70.5)	64.3 (54.4–74.2)	0.8176
Food/Shelter	63.9 (60.2–67.6)	62.1 (53.3–70.9)	62.6 (53.0–72.3)	56.8 (44.8–68.8)	69.3 (59.7–78.9)	64.8 (55.1–74.5)	0.8449
Healthcare	54.8 (51.0–58.6)	58.0 (49.2–66.9)	53.4 (43.3–63.4)	46.9 (35.1–58.6)	49.3 (38.7–59.8)	50.1 (39.4–60.9)	0.9235
Community Safety	57.1 (53.4–60.9)	55.6 (46.7–64.5)	55.6 (45.6–65.5)	49.4 (37.6–61.2)	52.3 (41.8–62.9)	55.5 (45.0–66.1)	0.7894
Transportation Services	40.3 (36.6–44.0)	40.6 (31.9–49.3)	34.9 (25.3–44.5)	41.6 (30.1–53.0)	35.1 (25.3–44.8)	35.3 (24.6–45.9)	0.5582

a. Applied survey weighting to ensure the sample reflects a representative analysis of disaster preparedness for the US population.

## Data Availability

The publicly available FEMA dataset can be found at https://www.fema.gov/about/reports-and-data (accessed on 4 January 2025).
